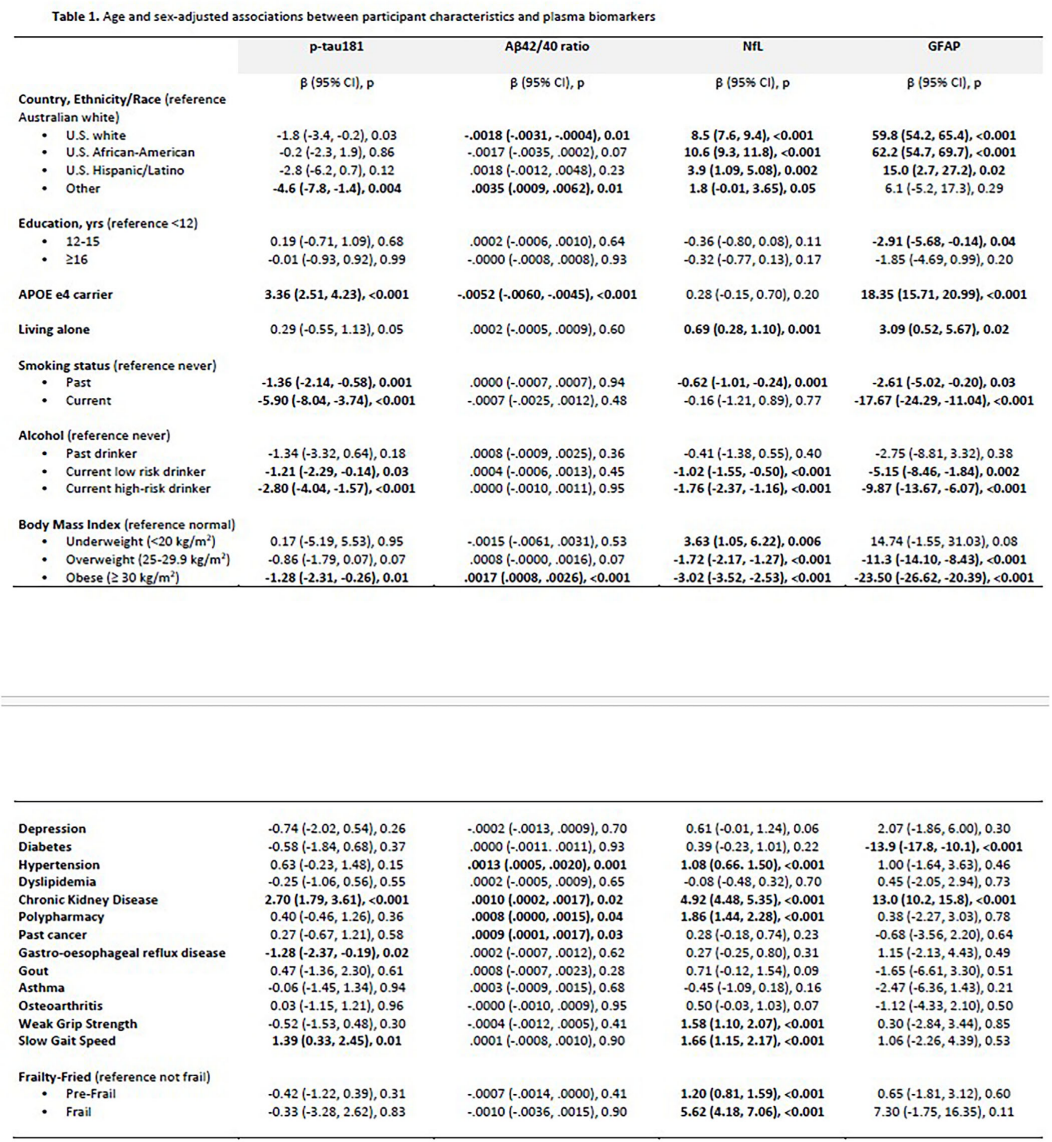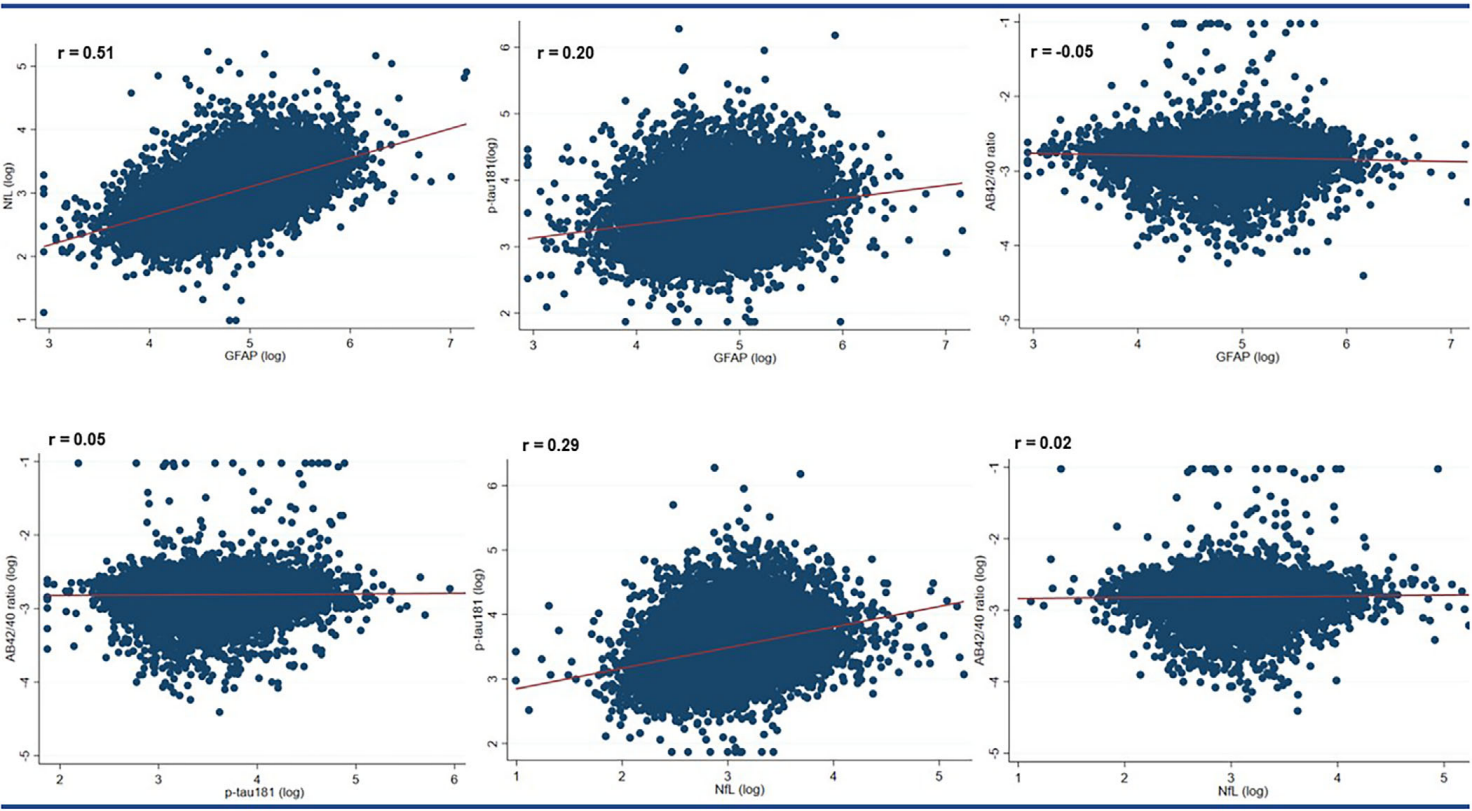# Plasma biomarkers of Alzheimer’s Disease and neurodegeneration in relatively healthy older community‐dwelling individuals

**DOI:** 10.1002/alz.095445

**Published:** 2025-01-09

**Authors:** Joanne Ryan, Anne Murray, Zimu Wu, Michelle M. Mielke

**Affiliations:** ^1^ Monash University, Melbourne, VIC Australia; ^2^ Berman Center for Outcomes and Clinical Research, Hennepin Healthcare Research Institute, Minneapolis, MN USA; ^3^ University of Minnesota, Minneapolis, MN USA; ^4^ Wake Forest University School of Medicine, Winston‐Salem, NC USA

## Abstract

**Background:**

Blood‐based biomarkers of Alzheimer’s Disease (AD) are ideally suited for use at the population level for screening, diagnosis, and for serial assessments to track disease progression. However, a number of critical knowledge gaps remain. Importantly, 1) these biomarkers have not been sufficiently examined in longitudinal studies of older community‐based populations without diagnosed dementia; and 2) it is unclear how participant characteristics such as sociodemographic characteristics and chronic conditions affect the clinical interpretation of these biomarkers.

**Method:**

In the U.S and Australia, we recruited 19,114 community‐dwelling individuals aged ≥70 years (U.S African‐Americans and Hispanics ≥65 years), without major cognitive impairment, cardiovascular disease and physical disability. Over 13,000 participants provided blood samples, and plasma amyloid‐beta (Aβ40 and Aβ42), phosphorylated tau (p‐tau 181), neurofilament light (NfL), and Glial Fibrillary Acidic Protein (GFAP) were measured [Simoa N4PE and Ptau181 v2 assays].

**Result:**

NfL levels were moderately correlated with the levels of GFAP (r = 0.51), and p‐tau181 had a low correlation with each of these biomarkers (r = 0.29 and 0.20 respectively). All other biomarkers were only weakly correlated with one another (Figure 1). Females (n = 7,218) had significantly lower levels of p‐tau181 (β: ‐0.117, p<0.001) but higher levels of NfL (β: 0.058, p<0.001), GFAP (β: 0.236, p<0.001), and Aβ42/40 ratio (β: 0.023, p<0.001) than males (n = 5,942). For both sexes, age was positively associated with levels of p‐tau181, NfL and GFAP, and negatively associated with Aβ42/40 ratio (Table 1). Ethnic and racial differences were also observed. After adjusting for age and sex, chronic kidney disease was associated with significantly higher levels of all plasma biomarkers, while alcohol consumption and obesity were associated with lower levels of p‐tau181, NfL and GFAP. Hypertension and polypharmacy were associated with significantly higher Aβ42/40 ratio and NfL, while frailty was only associated with higher NfL. Depression, dyslipidemia, gout, asthma and osteoarthritis, were not strongly associated with any biomarkers.

**Conclusion:**

Plasma biomarkers of AD and neurodegeneration appear to vary across sociodemographic characteristics, and might be directly influenced by certain lifestyle factors and chronic conditions. These findings highlight the importance of considering these factors when interpreting biomarker levels in regards to AD risk.